# Research progress of dissolving microneedles in the field of component administration of traditional Chinese medicine

**DOI:** 10.3389/fphar.2025.1623476

**Published:** 2025-08-13

**Authors:** Yuting Yang, Tingting Zhang, Baoshuo Niu, Xiangyu Wang, Liting Liu, Pengju Zhu, Fanda Meng

**Affiliations:** ^1^ Department of Cardiology, The First Affiliated Hospital of Shandong First Medical University & Shandong Provincial Qianfoshan Hospital, Shandong Medicine and Health Key Laboratory of Cardiac Electrophysiology and Arrhythmia, Jinan, China; ^2^ School of Clinical and Basic Medical Sciences, Shandong First Medical University & Shandong Academy of Medical Sciences, Jinan, China

**Keywords:** dissolving MNs, traditional Chinese medicine, transdermal administration, MNs preparation, skin restoration, Analgesia and anti-inflammation

## Abstract

In recent years, dissolving MNS technology, as an emerging transdermal drug delivery technology, has shown unique advantages and broad application prospects in the fields of transdermal drug delivery, transcutaneous immunity, beauty and skin care, food testing, and disease diagnosis. The active ingredients of traditional Chinese medicine have shown remarkable efficacy in treating various diseases. However, the mode of administration of traditional Chinese medicine (TCM) limits its potential for clinical application and promotion to a certain extent. The combination of dissolving MNS technology and transdermal administration of traditional Chinese medicine can not only simplify the application process of traditional Chinese medicine, but also promote the modernization process of traditional Chinese medicine and realize the “reduction,” “toxicity reduction,” and “efficiency increase” of traditional Chinese medicine. This article reviews the advances in the preparation and application research of dissolving MNS in traditional Chinese medicine. It provides a reference for further exploring the development and clinical application of efficient soluble MNS in traditional Chinese medicine.

## 1 Introduction

**FIGURE 1:**
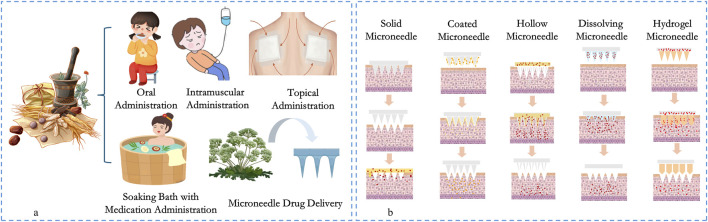
The administration method of traditional Chinese medicine **(a)** and the delivery modes of various types of herbal microneedles **(b)**.

Traditional Chinese medicine (TCM) can regulate related factors, impede signaling pathways and inhibit the growth of microorganisms, which can activate and support the body’s immune system, protect tissues or organs and enhance the body’s ability to resist and repair immune-related damage, acting as an antiviral, antimicrobial and anti-inflammatory agent (Huang et al., 2023; Zou et al., 2023; Xue et al., 2024). These are mainly attributed to the active substances such as alkaloids, flavonoids, polysaccharides, saponins, tannins, and polyphenols in Chinese herbal medicine, and TCM therapy has a broad medical prospect. However, the traditional modes of administration of TCM, including oral administration, transdermal administration, subcutaneous injection, intramuscular injection, and acupoint injection (Homayun et al., 2019; Yuan et al., 2025b), have certain limitations, which, to a certain extent, limit the continuous innovation and wide application of TCM. Oral administration inevitably faces the hepatic first-pass effect (Xue et al., 2014; Wang et al., 2017; Ehambarampillai and Wan, 2025), and its bitter taste reduces patient compliance, resulting in unsatisfactory drug delivery (Zheng et al., 2018). In addition, the traditional way of decocting TCM makes it difficult to precisely control the dosage of active ingredients, leaving a risk of overdose and potential liver or biliary injury (Chen et al., 2011; Shen et al., 2019; Jin et al., 2024). Traditional methods of transdermal drug delivery, such as smearing, applying, washing, and bathing, are difficult to penetrate the natural barrier of the skin’s stratum corneum, resulting in low drug bioavailability and unsatisfactory efficacy, as well as a waste of herbal resources (Paudel et al., 2010). What’s more, injection drug delivery methods such as subcutaneous injection, intramuscular injection, acupoint injection, and intravenous injection are not only accompanied by pain, but also consume a large amount of medical resources during the treatment process and have a long treatment cycle, which limit their popularity among patients (Li et al., 2018; Usach et al., 2019). Figure 1a shows the administration method of traditional Chinese medicine.

Microneedles (MNs) technology is an innovative means of physically facilitating penetration and consists of multiple micron-sized fine tips fixed in an array on a base. MNs are typically between 25 and 2000 μm in length. For example, the hollow MNs used in some studies are 900 microns long, while others are up to 500 microns in height (Aldawood et al., 2021). MNs’ transdermal drug delivery technology enables painless targeted drug delivery (Mdanda et al., 2021). When the MNs patch is applied to the skin surface, the tip of the MNs can penetrate the skin’s stratum corneum and form microporous channels that deliver drug molecules directly to the epidermis or dermis, allowing the drug to act locally in the affected area or to be distributed throughout the body via the circulatory system, thus enabling efficient transdermal drug delivery (Olatunji et al., 2013; Larrañeta et al., 2016). MNs’ patches are designed to be compact and easy to use and carry, due to the length of the needle body being at the micrometer level, patients only feel slight pain or even no pain during the process of use, which significantly improves patients’ drug compliance (Kaur et al., 2014; Ita, 2015; Avcil and Çelik, 2021). The novel drug delivery method combining MNs technology and active ingredients of drugs can effectively avoid the first-pass effect of the liver and gallbladder and break through the natural barrier of the skin stratum corneum, significantly improving the bioavailability of drugs.

The targeted transdermal drug delivery method that combines MNs technology with TCM components shows significant advantages compared with TCM delivery methods. According to the principle of *in vivo* drug release and the characteristics of MNs, they can be classified into the following five categories (Nguyen and Nguyen, 2023): Solid Microneedles (SMNs), Coated Microneedles (CMNs)、 Hollow Microneedles (HMNs), Dissolving Microneedles (DMNs), and Hydrogel Forming Microneedles (HFMNs). Figure 1b shows the drug delivery modes and characteristics of MNs. Each type of MN has unique advantages, while DMNs are widely favored by researchers due to their easy development process, convenient drug delivery modes, and stable drug loads (Aldawood et al., 2021; Maia et al., 2025). Among the transdermal delivery of herbal medicines, DMNs are the most widely used, more intensively researched, and have the greatest potential for clinical application.

A biodegradable polymer matrix is the material for developing DMNs of TCM. When MNs act on the organism, the polymer matrix will gradually degrade, releasing the active drug ingredients to exert the therapeutic effect. Through the precise control of MNS development technology and drug loading technology, it can realize the quantitative drug loading and slow release of drugs (Aldawood et al., 2021). Selection of a polymer matrix with appropriate drug concentration for loading into microneedles enables quantitative drug loading, enabling precise calculation of the amount of herbal medicine to be administered, thus reducing the drug dose and its toxic side effects (He et al., 2024). At the same time, by controlling the degradation rate of the polymer matrix, a slow release of the drug can be achieved, so that the body can maintain long-term effective blood concentration and prolong the duration of drug action (Ahmed Saeed Al-Japairai et al., 2020; Meng et al., 2024). Based on the advantages of TCM-DMNs in the field of transdermal drug delivery of TCM, this paper will focus on their application in the treatment of diseases and the development method, aiming to provide a reference for further research and development of TCM-DMNs and their clinical application.

## 2 Development of dissolving microneedles of TCM

DMNs are developed by mold casting methods, lithography, droplet blowing, spray deposition, and 3D printing (Zhuo et al., 2025). Out of these methods, the mold casting method has gained the highest usage rate due to its easy procedure and flexible operation. Polydimethylsiloxane (PDMS) is a commonly used material for Dissolving Microneedle Molds (DMN Molds). In addition, materials such as polytetrafluoroethylene (Baek et al., 2021) and epoxy resin (Singh et al., 2025) have been used to make DMNs molds.

### 2.1 Substrate materials for dissolving microneedles

Polymeric materials commonly used for DMNs matrices in the mold casting method include Polyvinylpyrrolidone (PVP), Hyaluronic Acid (HA), Polyvinyl alcohol (PVA), Carboxymethyl Cellulose (CMC) and its sodium salt (CMC-Na) Polyethylene Glycol (PEG), Chitosan (CS), Polylactic Acid (PLA), Copolymer of Methyl Vinyl Ether and Maleic Anhydride (Zhuo et al., 2025). All of these materials are biodegradable, non-toxic, and non-hazardous. Since using a single matrix material is often difficult to achieve the desired results, a composite matrix is used as a component of the tip and backing of DMNs to ensure that the microneedles are sufficiently resilient and firm.

### 2.2 Development of microneedles by the casting method

**FIGURE 2:**
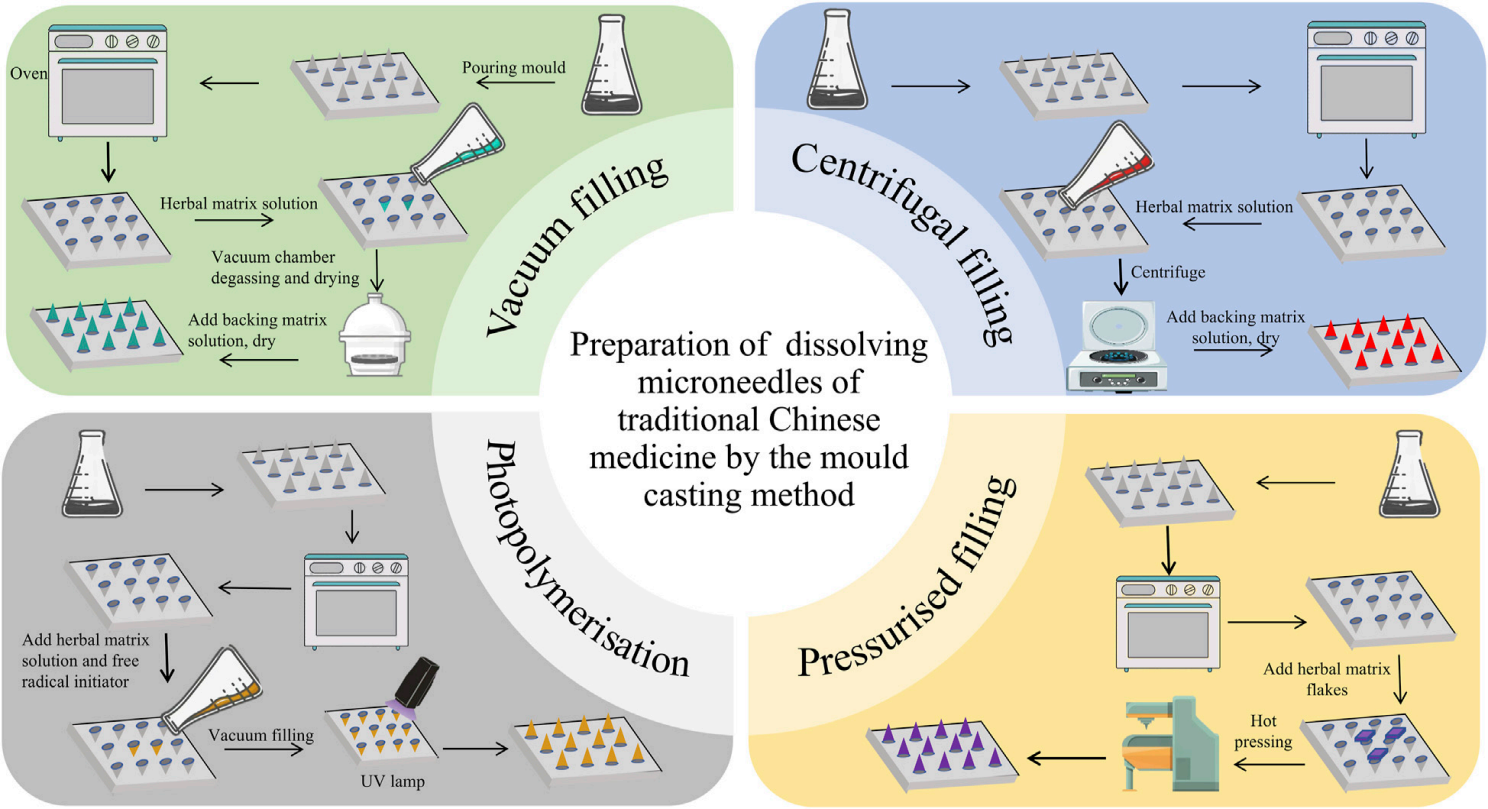
Development of dissolving microneedles of traditional Chinese medicine by the mold casting method.

The filling methods of the mold casting method mainly include the vacuum filling method, centrifugal filling method, pressurized filling method, and photopolymerization method. Among them, the more widely used methods are the vacuum filling method and centrifugal filling method because of their convenient operation and ability to remove air bubbles in the solution to ensure microneedle performance. The four mold filling methods are shown in Figure 2.

#### 2.2.1 Vacuum filling method

The vacuum filling method uses materials such as PDMS to make microneedle molds to ensure that the surface of the molds is smooth and flawless, and that the MN arrays have a precise structure and clear pinholes. The TCM matrix solution mixed with an appropriate amount of soluble polymers (e.g., PVP, HA, etc.) is stirred well to form a solution suitable for pouring. After pouring the solution into the mold, it is placed in a vacuum drying oven and evacuated under - 0.1 MPa vacuum to remove air bubbles to ensure the solution fills the pinholes sufficiently to avoid affecting the molding quality. Subsequently, the mold is placed in a drying oven at 40 °C–60 °C to allow the solvent to evaporate, the polymer to solidify, and the microneedle structure to form, with a drying time generally ranging from several hours to tens of hours. This method removes air bubbles by vacuuming and ensures a homogeneous internal structure of the MNs, which improves the mechanical strength and drug loading while helping the solution to penetrate sufficiently to ensure the molding rate and dimensional accuracy.

#### 2.2.2 Centrifugal filling method

The centrifugal filling method also uses materials such as PDMS to make microneedle molds to ensure that the quality and structure of the molds meet the requirements. The herbal matrix solution mixed with soluble polymer is stirred well to obtain a solution suitable for centrifugal pouring. After pouring the solution into the mold, it was fixed on a centrifuge and centrifuged at 1000–3000 r/min for 5–15 min, using centrifugal force to make the solution rapidly fill the pinhole and expel air bubbles. After completing centrifugation, the mold is placed in a 40 °C–60 °C drying oven to allow the solvent to evaporate and the polymer to cure, forming the MNs structure. This method uses centrifugal force to improve pouring efficiency and molding quality, reduce air bubble defects inside the MNs, and enhance MN performance.

#### 2.2.3 Photopolymerization method

The photopolymerization method uses photosensitive mold materials (e.g., photosensitive resin) to make microneedle molds, the surface of which has been treated with a special photosensitive treatment that allows polymerization reactions to occur under light. The herbal matrix solution mixed with the photoinitiator is stirred well to form a photosensitive solution. After pouring the photosensitive solution into the mold, it is placed under UV or visible light irradiation. The light intensity and time are determined according to the nature of the photoinitiator and the thickness of the mold, and the photoinitiator decomposes to produce free radicals, which trigger the polymerization reaction to form a solid microneedle structure. After completing photopolymerization, the MNs are removed, cleaned, and dried to remove the residual solution and impurities, and finally obtain the TMC-DMNs. This method of rapid curing and shaping by photoinitiated polymerization reaction can be carried out at lower temperatures to avoid high temperatures from destroying the components of TCM and retaining the activity of TCM, while accurately controlling the size and shape of the microneedles to develop microneedles with complex structures.

#### 2.2.4 Pressurised filling method

The pressurized filling method uses pressure-resistant mold materials (e.g., metal or rigid plastic) to make MN Molds with good sealing properties. The herbal matrix solution mixed with a soluble polymer is stirred well to obtain a solution suitable for pressurized pouring. The solution is poured into the mold and then sealed, and 0.1–0.5 MPa pressure is applied through pressurized equipment (e.g., air pump or hydraulic press), so that the solution is pressed into the needle holes and air bubbles are discharged under pressure. Once the pressurization is complete, the mold is placed in a 40 °C–60 °C drying oven to allow the solvent to evaporate and the polymer to cure, forming the microneedle structure. This method improves the casting efficiency and molding quality by applying pressure, reduces the defects of air bubbles inside the MNs, enhances the performance of MNs, and completes the casting process in a shorter period to promote the production efficiency.

## 3 Application of dissolving microneedles in TCM

In recent years, the combination of TCM components and DMNs technology (TCM-DMNs) for drug delivery has demonstrated significant application value and broad prospects for development in various fields. Currently, dissolving microneedles containing herbal components have shown positive effects in multiple aspects, such as treating skin diseases, promoting wound healing, providing pain relief and anti-inflammatory effects, fighting cancer, improving health management, and enhancing the immune system. As a new route of transdermal drug delivery, the combination of MNs technology and TCM components has been first explored for research and application in the treatment of skin-related diseases, such as hyperpigmented dermatosis (Chen et al., 2025), wound healing (Ji et al., 2024), and scar repair (Liu Y. et al., 2024). In addition, given the abundance of analgesic and anti-inflammatory active ingredients in TCM, research on harnessing TCM-DMN technology for drug delivery to alleviate painful diseases such as arthritis (Wang X. et al., 2025) and headache has entered the clinical trial stage and achieved positive feedback on clinical effects. In addition, MC-DMNs have also made progress in other disease areas, especially in the areas of ‘fat loss’ and “anti-cancer,” which has generated great public interest.

### 3.1 Treatment of skin diseases

#### 3.1.1 Improvements of skin pigmentation

TMC-DMNs technology has proven effective in promoting skin wound healing and improving skin diseases such as melasma (Chen et al., 2025). Chloasma is a hyperpigmented skin condition that is more common in women and harms the psychological and social life of patients, especially those who are more concerned about their outward appearance (Passeron and Picardo, 2018). Despite various clinical treatments for melasma and, beauty institutes and skincare companies marketing their products as having therapeutic benefits, achieving a complete cure for melasma remains notably challenging (Srivastava et al., 2025). Research on the synergistic treatment of melasma by TCM-DMNs has made positive progress. It is currently believed that resveratrol plays a role in reducing pigmentation deposits and has whitening and anti-aging effects by suppressing tyrosinase catalytic activity, tyrosinase gene expression, tyrosinase protein maturation, autophagy, and other processes to attenuate cellular melanin synthesis. Avcil and other researchers (Avcil et al., 2021) used the air-liquid blowing method to develop HA-based microneedles (HA-MNs) and integrated resveratrol and tranexamic acid to target the skin discoloration phase. They evaluated the tolerance and efficacy of HA-MNs with the mixtures targeting melasma in subjects. The final results showed that 95% of the subjects exhibited visible color-reducing changes, proving that drug-carrying HA-MNs can effectively treat melasma. Glabridin (GLA) (Chen et al., 2016; Pan et al., 2023) is an isoflavone isolated from the roots of Glycyrrhiza glabra, which inhibits tyrosinase activity and downregulates the transcription and protein expression of melanogenesis-related factors. Yan et al. (2023) developed DMNs prepared with HA/PVA/PVP and loaded with drugs such as GLA. They verified the significant antioxidant and inhibitory effects of DMNs on tyrosinase activity through melanin content assays, zebrafish experiments, and clinical trials.

Combining the pharmacological activity of TCM with the transdermal delivery advantage of DMNs, TCM-DMNs can effectively inhibit melanin production and improve skin pigmentation. This not only demonstrates remarkable efficacy in treating chloasma and other skin disorders but also offers a novel strategy and approach for clinical management.

#### 3.1.2 Acne treatment

Acne is one of the three most common skin diseases among the general population, primarily affecting young people aged 12–25, with its prevalence continuing to rise in late adolescence. Acne is primarily caused by the excessive growth of Propionibacterium acnes, usually concentrated on the face, neck, chest, and other such areas, affecting appearance and causing social distress and psychological pressure for patients (Malik and Kaur, 2018; Arsenie et al., 2020). Traditional treatment methods for acne include topical medications such as ointments and gels, but their therapeutic efficacy is limited. MN transdermal delivery can effectively solve this problem.

Sodium houttuyfonate (SH) is a bioactive compound derived from the TCM Sodium Houttuynia cordata, exhibiting antibacterial, anti-inflammatory, oxidative stress-suppressing and cardioprotective effects (Shingnaisui et al., 2018; Liu et al., 2021). Xin et al. (2024) selected SH as the key therapeutic agent for addressing acne vulgaris. Based on the high biocompatibility, antibacterial efficacy, excellent water solubility, and pH-responsive characteristics of N, O-carboxymethyl chitosan (NOCC), they developed pH-responsive TCM-DMNs by cross-linking SH and NOCC through the Schiff base reaction, allowing SH to release at the optimal pH for antibacterial effect. At the same time, it was proved that SH-NOCC directly inhibits the growth of Propionibacterium acnes and the occurrence of inflammation by suppressing the NF-κB/NLRP3 signaling pathway, achieving the effect of treating acne. Dendrobium officinale polysaccharide (DOP) is the main active component of the TCM. It exhibits immunomodulatory, anti-inflammatory, and antioxidant effects (Guo et al., 2025; Liang et al., 2025), as well as high stability, biocompatibility, and biodegradability. Wang et al. (2025c) developed and verified that the DMNs with DOP as the matrix have good mechanical and transdermal properties. Then, they combined antibacterial chrysanthemum flavonoids (CCF) and Poly (lactic-co-glycolic acid) (PLGA) nanoparticles prepared from the anti-acne drug adapalene (Adap) to fabricate drug-loaded micro-needles (DOP/CCF/PLGA@Adap-MN). to fabricate drug-loaded MN(DOP/CCF/PLGA@Adap-MN). Through the anti-inflammatory and antioxidant effects of DOP, the effective inhibition of Propionibacterium acnes growth by CCF, and the release of Adap by PLGA@Adap NPs, the three components work together to treat acne. In addition, Xing et al. (2022) prepared drug-loaded MN (AZA-MN) using HA and PVP as the MN matrix and azelaic acid (AZA) as the active ingredient. AZA-MN can quickly eliminate acne abscesses, accelerate the healing of skin lesions, and effectively treat acne.

TCM-DMNs enhance the efficiency and efficacy of treating acne, significantly curb the growth of Propionibacterium acnes and the occurrence of inflammation, demonstrate high biocompatibility and stability, and thus present an innovative solution for acne treatment.

#### 3.1.3 Psoriasis treatment

Psoriasis is a typical immune-mediated inflammatory skin disease, with a prevalence ranging from 0.14% to 1.99%. The occurrence of psoriasis is due to the activation of various inflammatory pathways mediated by immune cells, which leads to abnormal proliferation of keratinocytes, resulting in thickened skin epidermis and the appearance of scales (Feng et al., 2025). TCM such as quercetin, curcumin, licorice, and saussurea contain various bioactive components. These components exert pharmacological effects ranging from immune regulation and anti-proliferation to suppression of angiogenesis, providing feasible treatments for individuals with psoriasis.

Quercetin (Aghababaei and Hadidi, 2023), a flavonoid with anti-inflammatory, anticancer, and antimicrobial properties, exhibits poor transdermal permeability, which restricts the bioavailability associated with traditional administration methods. Paleco et al. (2014) significantly improved the transdermal delivery efficiency of quercetin by combining emulsification and ultrasonic treatment with DMNs technology, finally verifying its effect on improving skin inflammation in a psoriasis-like dermatitis model. Methotrexate is currently considered the most effective drug for treating psoriasis. Its oral administration not only has side effects such as liver toxicity, nausea, vomiting, and leukopenia, but also has a low biological utilization rate. Zhao et al. (2025b) reported that a series of micro-needles composed of cross-linked gelatin methacrylate and puerarin could be used for continuous intradermal administration of methotrexate to treat psoriasis. By loading methotrexate and the protective and alleviating active ingredient of puerarin, which has a protective effect on atopic dermatitis, onto the micro-needles, effective treatment of psoriasis was achieved. It was proven that there is a definite synergistic therapeutic effect between methotrexate and puerarin in treating psoriasis. Furthermore, ginsenoside Rg3 has also been shown to be effective in treating psoriasis. However, the keratin layer barrier of the skin affects the drug delivery performance through percutaneous non-invasive administration. Huang et al. (2022) reported a lipid-based drug delivery system with anti-inflammatory and immune-regulating functions, incorporating cholesterol-free liposomes loaded with ginsenoside Rg3 and integrated into HA-DMNs,. The study demonstrated that this system exhibits higher drug bioavailability, longer *in vivo* retention time, and greater efficacy compared with lipid-based percutaneous administration alone. Wu et al. (2025) constructed a mitochondrial-targeted curcumin derivative MN (Cur-TPP@Mil MN) for the treatment of psoriasis. Their study showed that the use of Cur-TPP@Mil MN reduced epidermal proliferation by 70% within 10 days in experimental animals and restored immune homeostasis.

Harnessing the anti-inflammatory, immune-regulatory, and epidermal proliferation-inhibiting properties of active ingredients from TCM to treat psoriasis, and combining these with the advantages of targeted sustained-release delivery via DMNs, offers an effective therapeutic strategy for the condition.

### 3.2 Skin repair

#### 3.2.1 Wound healing

*Centella asiatica* (AS), a traditional Chinese medicine, has been widely used to promote wound healing (Somboonwong et al., 2012; Arribas-López et al., 2022). Historically, AS was used to make decoctions for internal consumption or external application to accelerate wound recovery. Researchers from the CHI J team (Chi et al., 2021) designed a dissolving microneedle patch of TCM containing AS. This patch was developed by mixing the fresh juice of tofu chai with AS solution and solidifying it in a mold. This microneedle patch not only preserves the effective active ingredients of TCM but also avoids the possible side effects of traditional chemical processing. Based on tofu wood and AS, microneedle patches have remarkable effects in promoting wound healing and perform outstandingly in multiple aspects, such as antibacterial, anti-inflammatory, collagen deposition, angiogenesis, and tissue reconstruction. Researchers Liu et al. (2024a) successfully developed composite microneedles containing Astragalus polysaccharide nanoparticles. This multifunctional system for drug delivery, based on the active ingredients of TCM, promotes wound healing by inhibiting the ROS/NF-κB signaling pathway (Liang et al., 2025) and regulating the polarization process of macrophages. Curcumin is a polyphenol compound extracted from turmeric, which exerts anti-inflammatory and antioxidant effects by eliminating reactive oxygen species and enhancing the antioxidant capacity of cells, effectively promoting wound healing (Song et al., 2022; Zhang et al., 2022; Weng et al., 2023). Xiao’s team (Xiao et al., 2024) designed a bilayer microneedle with a tip made of sericin methacrylate (SilMA), metal-organic framework (MOF) Bi-PCN-222, along with curcumin, and a base layer made of PVA. MOF hydrogel has superior antibacterial properties. Combined with the assisted anti-inflammatory effect of curcumin, it greatly improves the efficiency of wound healing. In addition, pH-sensitive fluorescent indicators were added to the PVA microneedle substrate. Real-time monitoring of fluorescence images and PH in a smartphone can effectively detect the degree of wound healing. These multifunctional TCM-DMNs have antibacterial, immunomodulatory, and antioxidant properties and can also promote the formation of new blood vessels. They demonstrate outstanding biocompatibility, which helps address the issue of antibiotic resistance and shows great potential in promoting the healing of diabetic wounds (Yadav et al., 2024).

The use of TCM-DMNs enables the drug components to directly act on the inflammatory area without affecting the patient’s daily life, while avoiding the waste of medical resources. However, the development process of these DMNs needs to be further optimized to improve their stability and drug loading.

#### 3.2.2 Scar repair

As a natural outcome of wound healing, the abnormal repair process of scars may lead to excessive tissue hyperplasia, resulting in hypertrophic scars. This kind of lesion is often accompanied by burning pain and itching, and the appearance is also unsatisfactory, seriously affecting the quality of the patient’s life (Nischwitz et al., 2020). At present, the clinical treatment methods for hypertrophic scars include drug therapy, stress therapy, laser therapy, hormone therapy, and radioactive element therapy (Mokos et al., 2017). However, these methods often have a long treatment cycle and are accompanied by discomfort, causing inconvenience to patients’ lives. The application of MNs technology offers a new possibility for treating hypertrophic scars. Transdermal drug delivery can exploit the effects of active ingredients more conveniently, thereby improving the quality of patients’ lives (Liu Y. et al., 2024).

AS has the effects of anti-fibrosis, anti-depression, anti-tumor, and anti-inflammation, which are helpful for scarless wound healing. However, the low permeability of the skin and poor solubility in water constitutes the main obstacles to its clinical application. Panax notoginseng saponin (PNS) (Zhi et al., 2021), a saponin component extracted from Panax notoginseng rhizomes, inhibits PI3K/AKT activation as well as knocks down TRPM7 in proliferative keloid fibroblasts, thereby inhibiting scar formation and effectively promoting scarless wound repair. In addition, due to its amphiphilicity and surface activity, PNS is also used as a stabilizer for nano-suspensions. Huang et al. (2024) prepared Asiaticoside - Panax notoginseng saponin - Nanocrystals (AS-PNS-NCs) by the solvent evaporation-ultrasound method, and further loaded them into DMNs to obtain AS-PNS-NCs-DMNs. In the rabbit ear keloid model, it was verified that it could inhibit hypertrophic scars. Shikonin (SHI) (Fan et al., 2019) is a naphthalene derivative extracted from Lithospermum. It effectively inhibits hypertrophic scars by inducing apoptosis of fibroblasts derived from hypertrophic scars, weakening extracellular collagen deposition, and inhibiting collagen deposition induced by TGF-β1 and cell-mediated contraction. Xiaoyu N’s team (Ning et al., 2021) developed HA-MNs carrying SHI, which demonstrated significant inhibitory effects on the viability and proliferation of fibroblasts in hypertrophic scars through *in vitro* experiments, and downregulated the expression of fibrosis-related genes. This transdermal drug delivery system significantly improves the bioavailability of SHI, providing a novel and convenient option for treating hypertrophic scars. The research team of Wu et al. (2021) designed a cyclodextrin metal-organic framework (CD-MOF) cross-linked with diphenyl carbonate, and then loaded it with quercetin (QUE), which significantly increased the solubility of QUE in water. After that, the CD-MOF carrying QUE was coated with the membrane of hypertrophic scar fibroblasts and then dispersed in microneedles made of white polysaccharides (BSP) for active targeted local treatment of scars to achieve uniform targeted drug delivery.

However, these studies mainly focused on *in vitro* and animal experiments, with relatively few studies on clinical applications. Therefore, there is still a great need for further research on the drug’s long-term stability and potential side effects. In addition, there are examples of TCM-DMNs being used for ulcer healing and diabetic wound repair. Application examples of dissolving microneedles for skin repair with traditional Chinese Medicine in Table 1.

**TABLE 1:** Application of dissolving microneedles for skin repair with traditional Chinese Medicine.

TCM	Active ingredient	Auxiliary materials	Solvent	Development method	length/μm	Application	Compound preparation	Literature
Astragalus	Astragalus polysaccharide	APB@Ber	Methanol	Molding	600	Diabetic wound healing	Nanoparticles	Liu et al. (2024a)
ginger	6-Gingerol	HA、CMC	DMSO	High-speed centrifugation	——	Diabetic wound healing	——	Yuan et al. (2025a)
Pilosa	Blood exhaustion	PVPK90 CS	methanol phosphate	Molding	——	Diabetic wound healing	——	Tang et al. (2024)
*Centella asiatica*	Asiaticoside, Panax notoginseng saponin	PVP-K90	DMSO	High-speed centrifugation	——	Hypertrophic scar	——	Huang et al. (2024)
Salvia miltiorrhiza	Tanshinone IIA	TSA, PVP	Ethanol	High-speed centrifugation	——	Hypertrophic scar	——	Zhan et al. (2023)
*Centella asiatica*	*Centella asiatica* acid	Grass ash solution	Ethanol	Molding	——	Wound healing	——	Chi et al. (2021)
Bletilla striata, *Centella asiatica*	Bletilla striata polysaccharide, asiaticoside	CS	Acetic acid	High-speed centrifugation	——	Wound healing	2-Hydroxypropyl-β -cyclodextrin	Lv et al. (2023)
Astragalus	astragalus polysaccharide	PVP K13-18	PBS	Molding	700	Oral ulcers	——	Zhang et al. (2025)

### 3.3 Treatment of osteoarthritis

Traditional Chinese medicine contains a variety of active ingredients with analgesic and anti-inflammatory effects, including alkaloids such as sophocarpine (Zeng et al., 2025), aconitine and scopolamine (Wu et al., 2025) as well as flavonoids such as quercetin, baicalin (Meng et al., 2025) and gingerol (Gupta et al., 2025) and also melittin (Carpena et al., 2020), tripterygium, resveratrol, etc. These components have limitations in traditional administration methods, resulting in unsatisfactory therapeutic effects. The application of MNs, especially local drug delivery, can achieve targeted therapy, directly acting on the inflamed and painful areas and avoiding the loss of drugs through the bloodstream, and thus taking effect quickly. Most of the TCM-DMNs are applied in the field of anti-inflammatory and analgesic treatment for osteoarthritis. Besides, there are also some other researches on the anti-inflammatory and analgesic effects of TCM-DMNs in other fields. Application examples of anti-inflammatory and analgesic applicationsof dissolving microneedles with traditional Chinese medicine in Table 2.

**TABLE 2:** Application of anti-inflammatory and analgesic applications of dissolving microneedles with traditional Chinese medicine.

TCM	Active ingredient	Auxiliary materials	Solvent	Development method	Length/μm	Application	Compound preparation	Literature
Coptis chinensis	Berberine	Aloe vera gel water chestnut starch		Molding	700	Anti-inflammatory and analgesic		Luan et al. (2024a)
The bark and leaves of the Iberian oak	Flavonoid quercetin	Lipid microparticles	Ethanol	——	——	Anti-inflammatory, anti-cancer, and anti-microbial	Lipid microparticles	Paleco et al. (2014)
Tripterygium	Triptolide	HA PVA		Two-step centrifugation	800	Osteoarthritis	Liposome—	Zhou et al. (2021)
Resveratrum veratrum	Resveratrol glycoside	CMC-Na,PVP-K30,HPMC,HA,PVA,HPC	——	Vacuum template	——	Acute gouty arthritis	Hydroxypropyl -β-cyclodextrin (HP-β-CD	Chen et al. (2020)
Green vine	Sinomenine hydrochloride	PVP CS	water	——	——	Anti-arthritis, anti-apoptosis, immunosuppression	——	Shu et al. (2020)
Astragalus membranaceus	Astragalus glycoside Astragalus polysaccharides	CTS-PVA	——	Dual-mode molding	——	Regulate immune function	——	Chen et al. (2024)

Although Aconitine (ACO) (Zhao et al., 2024) is often used to treat inflammation, such as pain and rheumatoid arthritis, its high cardiovascular toxicity limits its application range. Team Guo (Guo et al., 2019) enhanced the transdermal delivery safety of ACO by using Nanostructured lipid carriers (NLCs), and then embedded ACO-NLCs in PVP-based DMNs, effectively improving the arrhythmia problem caused by ACO. Researchers from Zhu et al. (2024) integrated 3-acetylaconitine liposomes (AAC-LIPS), oyster polysaccharides (ORP), and PVP into DMNs (AAC-ORP-DMNs) to solve the difficulty that AAC is insoluble in water. ORP exhibits better anti-inflammatory and antioxidant activities, enhances the activity of AAC, relieves pain in the spared nerve injury (SNI) model, and can be used for a long time.

Rheumatoid Arthritis (RA) is a chronic autoimmune disease that is difficult to cure. As the disease progresses, the teratogenicity rate of patients will increase significantly. Tripterygium (Shan et al., 2023) has the effects of dispelling wind and dampness and alleviating pain by unblocking the meridians. It is one of the traditional Chinese medicines used to treat RA. However, its main active ingredient, Triptolide (TP), has poor water solubility and is eliminated quickly in the body. Long-term use may cause toxicity and side effects on multiple organs. The Li et al. (2024a) developed DMNs carrying TP (TP-MNS) to locally deliver TP to the joint, thereby enhancing TP penetration and reducing adverse reactions such as hepatotoxicity and nephrotoxicity. Meanwhile, the team drew on traditional Chinese acupuncture therapy - acupoint administration [such as moxibustion (Tao et al., 2021; Jia et al., 2022; Fu et al., 2024)], and innovatively studied whether DMNs could simulate acupuncture needles to achieve the effect of “acupuncture and drug combination”. At the end, TP-MNS effectively alleviated the inflammatory response, joint swelling, and bone erosion in adjuvant-induced arthritis rats, proving that TP-MNS is a safe and convenient transdermal drug delivery method. Luan et al. (2024b) developed a photoresponsive and pH-responsive microneedle loaded with TP and paeoniflorin. Meanwhile, TP was loaded using polydopamine-MXene (P-MXene) to improve the embedding efficiency of TP. Resveratrol (Meng et al., 2021), an analgesic and anti-inflammatory drug, has a low bioavailability in clinical administration due to its poor water solubility. The nanocrystalline resveratrol DMNs (Res NC DMNs) developed by the Diao et al. (2024) demonstrated excellent stability and skin penetration performance. Combined with nanotechnology, they improved the solubility and bioavailability of resveratrol, providing a new idea for integrating nanomedicine and transdermal drug delivery systems. Application examples of dissolving microneedles with traditional Chinese medicine in the treatment of rheumatoid arthritis in Table 3.

**TABLE 3:** Application of dissolving microneedles with traditional Chinese medicine in the treatment of rheumatoid arthritis.

TCM	Active ingredient	Auxiliary materials	Solvent	Development method	length/μm	Application	Compound preparation	Literature
Aconite root	Aconitine	AIBN, PVP	——	Purple diplomatic communication	350	RA	Nanostructured lipid carriers	Guo et al. (2019)
Tripterygium	Triptolide	HA, PVP, PVA	Ethanol	Two-step casting process	1200	RA	——	Li et al. (2024a)
Resveratrum veratrum	Resveratrol	Res NC,HA,PVP	——	Vacuum pouring	500	RA	Nanocrystals	Diao et al. (2024)
Ma Qianzi	Strychnine	CS-PVP PVA	water	Two-step centrifugation	——	RA	——	Song et al. (2021)
Chili pepper	Capsaicin	HA, PVP		Wire drawing lithography	−600 ± 10	RA	——	Dangol et al. (2016)
Bee venom	Melittin	HA	MeHA	Microforming	700	RA	——	Du et al. (2021)
Peach tree	Peach gum polysaccharide	Tetracycline-loaded nanoparticles, PVA, HA	——	Vacuum micro-template	——	RA	Nanoparticles	Hu et al. (2022)

### 3.4 Anticancer

TCM has been widely used in China for thousands of years. By acting on multiple signaling pathways and molecular targets related to cancer, it achieves anti-cancer therapeutic effects with few side effects (Xiang et al., 2019; Sun et al., 2021; Yao et al., 2021). DMNs combined with TCM have many potential research directions in the anti-cancer field. DMNs have shown advantages in the field of anti-cancer drug delivery due to their targeted drug delivery and reduction of systemic side effects (Moreira et al., 2019). Meanwhile, the components of TCM have a mild therapeutic effect on cancer and are less toxic to oneself. The use of TCM-DMNs for drug delivery has an excellent development prospect in treating cancer. TCM-DMNs have been applied in the fields of anti-ovarian cancer, melanoma, and squamous cell carcinoma, etc. Application examples of the anti-cancer application of dissolving microneedles with traditional Chinese medicine in Table 4.

**TABLE 4:** Application of the anti-cancer application of dissolving microneedles with traditional Chinese medicine.

TCM	Active ingredient	Auxiliary materials	Solvent	Development method	length/μm	Application	Compound preparation	Literature
Turmeric	Elemene	ICG, PDMS		Two-step casting process		Melanoma	——	Tian et al. (2025)
Turmeric	Curcumin	HA, CMS-Na		Molding	——	Melanoma		Cheng et al. (2020)
Turmeric	Curcumin	PVA Sodium dodecyl sulfate	Ethanol	Two-step centrifugation	900	Melanoma	Nanoparticles	Vora et al. (2018)
Turmeric	Curcumin analogues	BSP, BDP, HA	DMSO	Ultrasonic casting	——	Oral squamous cell carcinoma	——	Ma et al. (2024)

Elemene (ELE) (Jiang et al., 2016) is a terpene compound extracted from the Wenyu of the Zingiberaceae family. It has many anti-tumor and therapeutic effects, but its insolubility and volatility limit its use in MNs. Tian et al. (2025) developed a two-layer MNs system through a two-step casting process. They used PDMS as the microneedle material, and embedded ELE in the backlayer of MNs. ELE diffused into the skin through the pores of PDMS to exert an anti-tumor effect. Meanwhile, ELE photosensitive MNS (ICG-ELE-MNs) were developed by combining with Indocyanine green (ICG). Under NIR light, the ICG in ICG-ELE-MNs converts light energy into heat energy and releases reactive oxygen species, promoting drug release. Furthermore, researchers verified through the melanoma mouse model that ICG-ELE-MNS can effectively promote ELE release and tumor treatment. Ginsenoside Rg3 has demonstrated significant anti-cancer activity in various cancer models and exerts its anti-cancer effects by regulating multiple signaling pathways (Xu et al., 2023). Yi et al. (2024) designed an MN composed of an RG3-methylacrylamide (GelMA) tip and a 2-hydroxy-2-methylpiphenyl-hydrogel base, which has good biocompatibility and drug carrying efficiency. It is used to directly deliver RG3 to the surface of ovarian tumors to exert anti-tumor effects. Researchers Zhao et al. (2025a) discovered that the active component of Hemiphyllum, clementine, can inhibit the growth of colorectal cancer by targeting the MAPK14 pathway. Combining it with DMN delivery technology can enhance the local drug delivery permeability and targeting. DMNs, with their ability to break through the skin barrier and directly deliver drugs to adjacent tissues of the tumor, can reduce the first-pass effect in the liver. The team of Ruan Shuyao (Ruan et al., 2024) constructed DMNs combined with functionalized CD-MOF and other nanocarriers to load hydrophobic TCM components such as curcumin, achieving stable drug delivery, enhancing anti-angiogenic and pro-apoptotic effects, and providing a technical reference for the composite system of TCM nanocarriers and MNs. In the future, this technology can be applied to the local treatment of breast cancer or melanoma, inhibiting the VEGF signaling pathway or the PI3K/Akt pathway to achieve an anti-cancer effect.

### 3.5 Health management

#### 3.5.1 Weight-loss

Obesity has been identified as one of the key factors affecting human health, and achieving “painless weight loss” has long been the goal pursued by researchers (Madigan et al., 2022). Multiple studies have shown that certain active ingredients in TCM can promote fat conversion and reduce body fat percentage (Akour et al., 2020). For instance, extracts from barley, Polygonum multiflorum, longan, Rhizoma Chuanxiong, lily bulb, and ginger all show significant fat-reducing effects (Hasani-Ranjbar et al., 2009). However, traditional drug administration methods often have difficulty achieving full contact between the drug and adipose tissue, which limits the exertion of the drug’s efficacy (Ashour et al., 2023). The TCM-DMNs, through transdermal drug delivery, let the drug directly act on the subcutaneous adipose tissue, thereby improving the therapeutic effect (Pan et al., 2025).

Capsaicin, as an effective phytochemical against obesity (Ma et al., 2024), has been encapsulated in clove oil and sodium caseate nanocarrier systems by researchers Seema Mudhol and Serva Peddha (2023), and transdermal delivery has been achieved through DMN patches. Studies have shown that the plasma bioavailability of capsaicin in nano-preparations is superior to that of native capsaicin, and the transdermal administration of DMN patches helps convert white adipose tissue (WAT) into brown adipose tissue (BAT), which is of great significance for the treatment of obesity. Peng et al. (2020) constructed a layer of black phosphorus, which was modified on the back of a DMN patch loaded with rosiglitazone (Rosi), thereby achieving a surgery-free method for rapid weight loss. Under near-infrared irradiation (NIR), Rosi can be effectively delivered without pain to the target adipose tissue, thereby achieving precise slimming. According to experiments in a high-fat-induced obese mouse model, the mice’s weight decreased by approximately 8%, while their waist circumference decreased by approximately 21% after 1 month. Furthermore, researchers Nayak et al. (2024) designed resveratrol nanostructured lipid carriers (NLC) and integrated these NLC into MN arrays to evaluate their anti-obesity activity in animal models. The research results show that after the successful delivery of resveratrol by DMNs, it demonstrates better weight loss and fat reduction effects compared with systemic administration. Caffeine has anti-obesity activity and no adverse effects (Zheng et al., 2004; Kobayashi-Hattori et al., 2005). However, due to the first-pass effect, the caffeine level in the plasma is uneven after oral administration. Moreover, in transdermal administration, the polymorphic transformation of caffeine from anhydrous form to hydrated form, which means the formation of caffeine crystals, limits the loading of transdermal administration (Nicoli et al., 2004). Researchers Dangol et al. (2017) developed HA-DMNs and loaded with caffeine. Because HA can inhibit the crystal growth of caffeine, it allows microneedles to carry a large amount of caffeine. The team confirmed the anti-obesity efficacy of CMP in the obese C57BL/6J mouse model induced by a high-fat diet, which can serve as an innovative approach for future caffeine-based clinical treatment.

#### 3.5.2 Hair regrowth

Hair loss is a common problem in daily life, affecting approximately 67% of men and 24% of women. Although hair loss does not impair physical health, it can cause patients to worry about their appearance, lose self-esteem, and experience depression, etc., (Williamson et al., 2001; Lin et al., 2016). Currently, hair loss is primarily treated with topical minoxidil, oral finasteride, low-intensity laser therapy, etc., to slow down hair loss and stimulate new hair growth. However, these treatment methods have some unavoidable side effects, such as affecting sexual function and lowering blood pressure (Irwig and Kolukula, 2011; Sanabria et al., 2023). Therefore, it is crucial to find new drugs and drug delivery methods for treating hair loss.

The plant belongs to the genus “Bletilla” of the Orchidaceae family and has significant medicinal value. The main bioactive component bletilla striata polysaccharide (BSP) exhibits hemostatic, anti-inflammatory, and hair growth-stimulating properties. In addition, BSP, used as a MN matrix, realizes sustained drug release due to its high viscosity. It can also act as a formulation agent to impart hardness to MNs and exhibits good biocompatibility, being harmless to the human body. Zhao et al. (2025c) used BSP and HA as microneedle matrices, loading plGA-encapsulated non-toxic hair regeneration drug tofacitinib (TFB) encapsulated nanoparticles (TFB@NP) to prepare TCM-DMN, achieving efficient and sustained TFB treatment for hair loss. Curcumin has anti-androgenic effects and can effectively improve hair loss caused by hormonal imbalance (Zhou et al., 2014; Srivilai et al., 2017). Moreover, zinc deficiency is also one of the factors that causes hair loss. Based on this, Yang et al. (2023) introduced curcumin and zinc ions into MOFs (ZnMOFs), and encapsulated them with γ-polyglutamic acid (γ-PGA) to develop dissolving ZnMOF-MN, which promotes hair growth by stabilizing and reducing androgen levels and inhibiting cell apoptosis. Platycladus orientalis leaf extract (PO-ex) inhibits 5α-reductase activity (Zhang et al., 2016), regulates the Akt/GSK3β/β-catenin signaling pathway (Zhang et al., 2013), and thereby promotes hair growth (Fu et al., 2023). Hong et al. (2025) extracted PO-ex using ethanol as a solvent, then loaded PO-ex onto the hydrogel cross-linked with hyaluronic acid methyl acrylate-hyaluronic acid (HAMA-HA), to prepare HA-MN (PO-ex MN). Moreover, PO-ex MN can activate the Wnt/β-catenin pathway related to wound repair and promote hair follicle growth. PO-ex MN is a potential treatment strategy for male hair loss.

TCM-DMNs have significantly fewer side effects than traditional hair loss treatments, with markedly improved drug delivery efficiency. They effectively regulate androgen levels and are expected to become a new solution for hair loss. However, these studies have only been conducted on mice and have not yet advanced to clinical applications.

#### 3.5.3 Sleep aid intervention

Insomnia is a common condition worldwide, characterized not only by difficulty falling asleep at night but also by daytime fatigue or sleepiness (Buysse et al., 2007). According to statistics, the global adult prevalence rate has reached 27.29% over the past 3 years (Wang J. et al., 2025). Insomnia seriously affects both physical and mental health and is closely related to the occurrence of cardiovascular diseases, diabetes, heart disease, and depression (Hertenstein et al., 2019; Rainer et al., 2023). Common treatment methods for insomnia include non-pharmacological and pharmacological treatments. Non-pharmacological treatments are divided into psychological therapy and physical therapy. Psychological therapy primarily focuses on insomnia cognitive behavioral therapy (van Straten et al., 2018), while physical therapy includes exercise, light therapy, and music therapy, etc., (Riemann et al., 2023). However, the therapeutic effect of non-pharmacological treatments is weaker than that of pharmacological ones. Pharmacological treatments mainly include benzodiazepine (BZ) and benzodiazepine receptor agonists (BZRA) (Liang et al., 2019), low-dose sedative antidepressants (Everitt et al., 2018), antipsychotics, dual orexin receptor antagonists (DORAs) (Kunz et al., 2023), antihistaminic drugs (Oyekan et al., 2021), melatonin, etc. However, Western medicine can cause side effects and dependence, and long-term use has great harm to the human body. TCM therapy includes taking TCM decoctions and acupuncture, which causes less damage to the human body with significant efficacy. However, taking TCM decoctions may cause pain to patients, and acupuncture therapy requires superb acupuncture skills, which limits the development of TCM therapy. Based on this, micro needles can be equipped with TCM and reduce the requirements for acupuncture point positioning, enhancing the efficacy of treating insomnia.

In response to the problems and limitations of existing sleep aid methods, He et al. (2024) developed a flexible TCM-DMNs for sleep aid intervention. Ziziphus jujuba spinosa kernel, polygala tenuifolia, albizia julibrissin flower, polygonum multiflorum stem, schizandra chinensis, *nelumbo nucifera* seed, and coptis chinensis were selected as the Chinese medicine components for the DMNs, which have the effects of calming the mind and promoting sleep. Later, HA was used as a carrier for TCM to develop TCM-DMNs. He et al. conducted a 3-week human experiment, applying the TCM-DMNs to the sleep aid point and the Yingtang point. Moreover, they compared the electroencephalogram signals on the first day and the last day, and found that the ratio of low-frequency brain wave energy to high-frequency brain wave energy significantly increased, indicating that the treatment of insomnia with TCM-DMNs was significant. TCM-DMNs have been applied in multiple treatment fields, and the field related to treating insomnia has not been deeply studied. He et al. combined TCM and DMNs for treating insomnia as an innovative attempt, expanding the application prospects of TCM-DMNs in the field of sleep aid.

## 4 The limitations and challenges of technology

### 4.1 The safety and acceptability of microneedle transdermal drug delivery technology

The tip height of DMNs is usually within the range of 200 μm–600 μm. This height can effectively penetrate the stratum corneum and enter the epidermis, achieving drug delivery without harming blood vessels or nerves. Therefore, the administration of DMNs causes only mild pain or no pain at all and will not cause severe nerve stimulation (Li Z. et al., 2024). This ensures the safety of the drug administration. Moreover, the materials currently used for DMNs are biocompatible, non-toxic, and degradable, thereby preventing harm to the human body from material toxicity. Compared with traditional injection drug administration, MN administration reduces patients’ fear of needles; compared with oral drug administration, MN administration greatly improves patient compliance and can effectively reduce the problem of not taking medication as prescribed due to large dosage, bitter taste of the medicine, etc. However, prolonged administration may lead to the accumulation of polymers in the body, resulting in granulation formation, local erythema, or accumulation in various organs. The long-term effects are not yet fully understood (Quinn et al., 2015).

### 4.2 The current challenges of the research

The limited surface area and needle length of DMNs restrict the drug loading volume of a single microneedle. Currently, research on TCM-DMNs tends to incorporate a single TCM component, with a lack of studies on loading compound medicinal agents onto DMNs. The volume limitation of the single-loaded drug load restricts the loading of compound drugs, and TCM-DMNs loaded with compound drugs will complicate the quantitative analysis of transdermal drug release and efficacy assessment. Moreover, the smooth delivery of lipophilic drugs is also a major challenge for TCM-DMNs in treating diseases. Zhao et al. (2025b) reported a type of MN that can generate bubbles, and a galingonine gelatin hydrogel carrying methotrexate was used for the treatment of psoriasis. The inclusion of effervescent agents (NaHCO_3_ and citric acid) in the backing layer of the MN enables it to rapidly generate bubbles (CO_2_) when in contact with the interstitial fluid of the skin, promoting the immediate separation of the needle body from the backing and entering the body, ensuring that all the drugs at the needle tip are delivered to the affected area; (Zhang et al., 2023) constructed an aerodynamic DMNs, which significantly improved the drug loading and delivery efficiency, with a drug loading of 1.35 times that of ordinary DMNs; (Tian et al., 2025) reported a Permeable polydimethylsiloxane MN for the delivery of TCM elemene. By combining volatile TCM with breathable PDMS, it promoted the transdermal release of lipophilic TCM. The researchers above have provided innovative solutions to address the bottlenecks of MN drug delivery technology, and this can be extended to other studies on TCM-DMNs.

### 4.3 Clinical trial status and regulatory obstacles

We used the keyword “microneedles” to search clinicaltrials.gov and identified a total of 124 clinical trials related to MNs. No ongoing or completed clinical trials of TCM-DMNs for disease treatment were found. Yang et al. (2025) study identified 57 relevant clinical trials in the last 5 years on microneedles; 10.5% (6/57) of them are in phase I, 15.7% (9/57) are in phase II, 10.5% (6/57) are in phase III, and 5.3% (3/57) are in phase IV. Most of the trials (47.4%, 27/57) are not applicable (NA), and 10.5% (6/57) of them are unknown. This suggests that clinical research in the field of MN drug delivery is expanding; however, research on TCM-DMNs still has a considerable distance to cover from laboratory studies to clinical trials. Zhuo et al. reported that MN administration may be regulated as an injection rather than a transdermal patch. Therefore, final MN products need to be evaluated for sterility and non-toxicity (Bauleth-Ramos et al., 2023). Furthermore, before being used in formal clinical applications, MNs require careful assessment of their safety, potential effects on the immune system, and the impact of polymer deposition in the human body during preclinical and clinical studies. However, there are currently no regulatory standards for evaluating the safety and toxicity of MNs, which hinders the approval process of completed MN preparations and limits their application and promotion. Improving regulatory standards related to MN preparations can facilitate clinical trials and subsequent promotion and application of TCM-DMNs.

## 5 Summary and prospect

The combination of TCM components and DMN technology for drug administration has shown great potential in treating various diseases. With different development methods, the effective delivery of multiple TCM components has been achieved, innovating the way of traditional Chinese medicine administration. DMN administration of TCM can ensure the precise quantification of drugs, solving the problem of excessive medication or unsatisfactory drug efficacy caused by inaccurate dosage in traditional administration methods (such as oral administration, application, etc.). In addition, the administration of TCM by MNs ensures that the dosage of the drug is within a safe range. For example, the safety of toxic active components of aconitine and tipterygium can be ensured by DMN percutaneous administration. And it also achieves the reduction of toxicity and enhancement of efficacy of toxic components by co-loading TCM with liposomes and nanoparticles during the development of microneedles, thus expanding the application prospects of TCM. MN drug delivery has realized the Westernization of TCM, realizing the potential of Chinese medicine in various fields of application. Expanding the usage scenarios of TCM and promoting the innovative development of the TCM industry.

Nowadays, most TCM components administered transdermally through DMN technology are single, small-molecule TCM components. The analysis and research of drug effects, the preparation of TCM-DMNs, and the evaluation of administration effects are relatively easier and more feasible. However, the methods of microneedle synergistic administration of compound TCM components or large-molecule TCM components, the proportion and drug concentration of each TCM component, and the efficacy of TCM-DMNs still need further research. In addition, although TCM-DMNs administration provides a more convenient way of drug delivery, whether the drug loading capacity of DMNs can meet the treatment needs of patients remains to be further studied. The improvement of DMNs development technology and the exploration of DMNs carrying TCM methods are the keys to achieving the wide application of this technology in clinical practice.

Although certain progress has been made in the technology of TCM-DMNs, there are still some challenges in carrying TCM with DMNs. Firstly, the complexity of the TCM components makes it difficult to predict and control their stability and release kinetics in DMNs. Secondly, the diversity of TCM and the demand for individualized treatment have put forward higher requirements for the design of DMNs and personalized drug administration strategies. In addition, the clinical application of TCM-DMNs still needs to overcome non-technical obstacles such as low patient acceptance and difficult regulatory approval.

Future research needs to optimize the development technology of DMNs, increase their drug loading capacity and stability, and explore more MN delivery systems for TCM components. In-depth research on the *in vivo* and *in vitro* release kinetics and pharmacodynamics of TCM-DMNs, as well as conducting more clinical trials, is important for promoting the practical application of this technology. With the continuous advancement of materials science, drug delivery technology, and clinical research, it is expected that DMN technology will provide strong support for the modernization and globalization of TCM, bringing more precise and personalized treatment plans. Especially in the fields that people are concerned about, such as anti-cancer, weight loss and fat reduction, and enhancing human immunity, the research on TCM-DMNs technology for drug administration has gradually emerged, showing vigorous prospects.

## Abbreviations

MNs, Microneedles; DMNs, Dissolving Microneedles; TCM, Traditional Chinese Medicine; PVA, Polyvinyl Alcohol; PVP, Polyvinylpyrrolidone; HA, Hyaluronic Acid; PDMS, Polydimethylsiloxane.
